# CDDO, an Anti-Inflammatory and Antioxidant Compound, Attenuates Vasospasm and Neuronal Cell Apoptosis in Rats Subjected to Experimental Subarachnoid Hemorrhage

**DOI:** 10.3390/cimb46050283

**Published:** 2024-05-13

**Authors:** William Winardi, Yun-Ping Lo, Hung-Pei Tsai, Yu-Hua Huang, Tzu-Ting Tseng, Chia-Li Chung

**Affiliations:** 1 Department of Neurosurgery, E-DA Hospital, Kaohsiung 82445, Taiwan; winardi930@yahoo.com; 2 School of Medicine, College of Medicine, I-Shou University, Kaohsiung 84001, Taiwan; 3 Department of Traditional Chinese Medicine, Kaohsiung Medical University Hospital, Kaohsiung 80708, Taiwan; pingping77114@gmail.com; 4 Division of Neurosurgery, Department of Surgery, Kaohsiung Medical University Hospital, Kaohsiung 80708, Taiwan; carbugino@gmail.com (H.-P.T.); cawaii7992@gmail.com (T.-T.T.); 5 Department of Neurosurgery, Kaohsiung Chang Gung Memorial Hospital, Kaohsiung 83301, Taiwan; newlupin2001@yahoo.com.tw; 6 Division of Neurosurgery, Department of Surgery, Kaohsiung Municipal Siaogang Hospital, Kaohsiung 81267, Taiwan

**Keywords:** SAH, CDDO, vasospasm, inflammation, apoptosis

## Abstract

Subarachnoid hemorrhage (SAH) is a type of stroke caused by bleeding into the subarachnoid space. SAH is a medical emergency and requires prompt treatment to prevent complications such as seizures, stroke, or other brain damage. Treatment options may include surgery, medication, or a combination of both. 2-Cyano-3,12-dioxoolean-1,9-dien-28-oic acid (CDDO), a compound with anti-inflammatory and antioxidant properties, is currently being investigated as a potential treatment for various diseases, including chronic kidney disease and pulmonary arterial hypertension. In this study, the effects of CDDO on rats subjected to SAH were evaluated. Male Sprague-Dawley rats were divided into four groups (*n* = 6/group): (1) control group, (2) SAH group, (3) SAH + low-dose CDDO (10 mg/kg injected into the subarachnoid space at 24 h after SAH) group, and (4) SAH + high-dose CDDO (20 mg/kg) group. CDDO improved SAH-induced poor neurological outcomes and reduced vasospasm in the basal artery following SAH. It also decreased the SAH-induced expression of proinflammatory cytokines such as TNF-α, IL-1β, and IL-6 in both the cerebrospinal fluid and serum samples as determined by ELISA. A Western blot analysis confirmed an increase in the p-NF-κB protein level after SAH, but it was significantly decreased with CDDO intervention. Immunofluorescence staining highlighted the proliferation of microglia and astrocytes as well as apoptosis of the neuronal cells after SAH, and treatment with CDDO markedly reduced the proliferation of these glial cells and apoptosis of the neuronal cells. The early administration of CDDO after SAH may effectively mitigate neuronal apoptosis and vasospasm by suppressing inflammation.

## 1. Introduction

Subarachnoid hemorrhage (SAH) is a medical emergency that often occurs due to rupture of cerebral aneurysms. SAH could result in severe brain injury. The development of cerebral vasospasm is a major complication following SAH.

Cerebral vasospasm develops in approximately 70% of patients between 3 and 14 days after SAH [1,2]. For decades, it has been considered the single and the most important cause of delayed cerebral ischemia and poor outcomes after SAH [3]. Cerebral vasospasm is thought to be caused by free radical production and subsequent lipid peroxidation, along with damage to the endothelium and apoptosis of endothelial cells [4].

Studies have shown a correlation among SAH, inflammation, and neuron apoptosis. Early brain injury after SAH involves signaling pathways related to neuronal apoptosis, inflammation, the oxidative stress response, and the protection of neurovascular units. Apoptotic cell death, which can occur in cerebral neurons or endothelial cells through the intrinsic or extrinsic pathways, has played a significant role in the prognosis in animal SAH models [5,6,7].

2-Cyano 2,3-dioxoolean-1,9-dien-28-oic acid (CDDO), also known as bardoxolone, is a synthetic derivative of oleanolic acid and a novel triterpenoid. CDDO derivatives have been studied for their potential therapeutic effects on inflammation and oxidative stress in multiple cell-signaling pathways, including nuclear factor erythroid 2-related factor 2 (Nrf2) and NF-κB. Yamauchi et al. reported that Nrf2 activators may attenuate brain injury after ischemia reperfusion in mice [8]. Another study revealed that CDDO derivatives could provide neuroprotection against ischemic injury by upregulating heme oxygenase-1 (HO-1), suggesting that enhancing HO-1 expression may be a legitimate strategy for the therapeutic intervention of stroke [9].

The correlation analysis conducted to date has suggested a strong negative correlation between CDDO and inflammation. However, the effect of CDDO on SAH-related pathology has not been reported. Therefore, this study was aimed to explore the potential therapeutic utility of CDDO in SAH.

## 2. Materials and Methods

### 2.1. Materials

CDDO, protease inhibitors, DAPI (F6057), and mouse anti-GFAP antibodies (G3893) were purchased from Sigma (St. Louis, MO, USA). Zoletil 50^®^ was obtained from Virbac (Carros, France). An antigen retrieval solution was acquired from DAKO (Carpenteria, CA, USA). Rabbit anti-Iba1 antibodies were purchased from Proteintech (10904-1-AP; Taipei, Taiwan). Goat anti-Rabbit IgG (H+L)-FAM (C04013) and Goat anti-Mouse IgG (H+L)-TAMRA (C04012) were obtained from Croyez (Taipei, Taiwan). Platinum ELISA kits were products of eBioscience (San Diego, CA, USA). DC Protein Assay Kit was acquired from Bio-Rad (Hercules, CA, USA). HRP-conjugated secondary antibodies (P36599A) and an ECL Western Blotting Detection kit (NEL122001EA) were from Millipore (Burlington, VT, USA) and PerkinElmer (Burlington, VT, USA), respectively.

### 2.2. Cell Culture

PC-12 cells (BCRC 60048) were maintained in an RPMI-1640 medium (Gibco, Waltham, MA, USA) enriched with 10% horse serum and 5% fetal bovine serum (both from Gibco), in a 5% CO_2_ humidified environment at 37 °C. To induce a neuronal phenotype, these cells were treated for 14 days with 50 ng/mL of nerve growth factor on culture flasks coated with Collagen IV. For experiments, PC-12 cells (1 × 10^4^ cells per well) were plated in 24-well plates and exposed to 500 μM H_2_O_2_. Concurrently, they were treated with varying concentrations of CDDO (ranging from 0 to 30 μg/mL) for 24 h. Following treatment, the culture medium was discarded. An MTT reagent was then added to each well at a concentration of 0.5 mg/mL and the cells were incubated for an additional 90 min. After this incubation, the medium was removed, and the purple formazan precipitate was solubilized with 200 μL of DMSO per well. Absorbance was measured using a Multiskan FC microplate photometer (Multiskan FC, Thermo Scientific, Waltham, MA, USA).

### 2.3. Animals

This study was carried out following a protocol sanctioned by the Institutional Animal Research Committee at Kaohsiung Medical University (IACUC 111018). Male Sprague-Dawley rats, each weighing approximately 350 g, were obtained from BioLASCO (Taipei, Taiwan). Upon arrival at the university’s vivarium, the rats underwent a minimum one-week acclimation period prior to experimentation. They were maintained in a facility with a 12 h light/dark cycle at a consistent temperature of 22.1 °C and 55% relative humidity. The rats had unrestricted access to standard feed and water throughout this study.

### 2.4. SAH Induction

The single-injection subarachnoid hemorrhage (SAH) model was employed in this study. Rats were initially anesthetized with an intraperitoneal dose of 40 mg/kg of Zoletil 50^®^, a combination of zolazepam and tiletamine hydrochloride. The rats’ heads were then secured in a stereotactic frame (Stoelting, Wood Dale, IL, USA). Using a 25-gauge butterfly needle, 0.3 mL of cerebrospinal fluid (CSF) was extracted from the cisterna magna. Subsequently, 0.1 mL/100 g of body weight of fresh, autologous, non-heparinized blood, harvested from the central tail artery, was infused into the subarachnoid space via the same needle connected to tubing. Post-injection, the rats were positioned ventrally for at least 30 min to ensure the proper distribution of the blood. Respiratory patterns were monitored carefully, with mechanical ventilation provided as needed. Once fully recovered, the rats were returned to their housing in the vivarium.

CDDO was dissolved in DMSO at a concentration of 50 mg/mL. All rats were used following randomization. There were four groups of 6 animals in this study: (1) control group (indicates vehicle, e.g., the solution that you used to dissolve CDDO); (2) SAH group; (3) SAH + low-dose CDDO (10 mg/kg injected into the subarachnoid space at 24 h after SAH) group; and (4) SAH + high-dose CDDO (20 mg/kg injected into the subarachnoid space at 24 h after SAH) group.

### 2.5. Neurological Behavioral Assessment

On day 7 following the administration of autologous blood, behavioral alterations in SAH rats were evaluated by an investigator who was not informed about the experiment’s specifics. The evaluation of motor functions was based on the rats’ ability to ambulate using their hind limbs and their performance on the placing/stepping reflex test, which involves dragging the hind paw dorsum along a surface edge. These assessments were scored using a previously established system [10] and are detailed in Table 1. The cumulative scores from the ambulation and placing/stepping reflex tests were combined to form the motor deficit index (MDI).

### 2.6. Tissue Processing

Upon the completion of the experiments, each rat was re-anesthetized to prepare for perfusion and fixation procedures. The thoracic cavity was accessed by inserting a No. 16 catheter into the left ventricle. Following the clamping of the descending aorta and the puncture of the right atrium, the brain was perfused with 180 mL of 2% paraformaldehyde followed by 100 mL of a 0.01 M phosphate buffer, maintaining a temperature of 36 °C and a perfusion pressure of 100 mmHg. A visual examination was conducted on the extracted brains to verify the presence of subarachnoid blood clots around the basilar artery (BA). The brains were then submerged in a fixative. Subsequently, the BAs were extracted from the brainstems, and the middle third of each artery was isolated for further processing. These arterial segments were embedded flat in paraffin, sectioned into 3 μm slices, and stained with Hematoxylin and Eosin for detailed histological evaluation.

### 2.7. Morphometric Assessment of BA

Three arterial cross-sections from the middle third of the basilar artery (BA) of each rat were examined by a trained technician who was unaware of the group assignments. The maximum vertical distance from the inner endothelial surface to the outer adventitial surface was recorded as the thickness of the BA. The cross-sectional area of the artery was determined using a computer-assisted morphometric analysis (Universal Imaging Corp., New York, NY, USA). To assess the extent of vasospasm, the average cross-sectional area of the BA from each rat was computed, and these averages were used to calculate the mean vasospasm values 48 h post-SAH.

### 2.8. Immunofluorescence

Following the deparaffinization and rehydration steps, brain samples embedded in paraffin were subjected to antigen retrieval by steam heating for 30 min in a DAKO antigen retrieval solution. The slides were then washed twice with Tris-buffered saline (TBS) and incubated overnight at 4 °C with mouse anti-GFAP and rabbit anti-Iba1 antibodies to label astrocytes and microglia, respectively. For the detection of neuronal apoptosis, sections were further incubated with mouse anti-NeuN and rabbit anti-cleaved caspase-3 antibodies for 16 h at 4 °C. After another set of double washes with TBS, the sections were incubated with Goat anti-Rabbit IgG (H+L)-FAM and Goat anti-Mouse IgG (H+L)-TAMRA for 90 min at room temperature. Following two additional washes in TBS, the slides were mounted using Fluoroshield^TM^ containing DAPI. Fluorescent images were acquired using an Olympus fluorescence microscope (U-RFL-T), and the fluorescence intensity and numbers were quantified using ImageJ software, version 1.44d (NIH).

### 2.9. Enzyme-Linked Immunosorbent Assay (ELISA)

Animals were sacrificed 7 days after SAH, and 100 μL of CSF was collected to determine protein levels of neuron-inflammatory cytokines IL-6, TNF-α, and IL-1β, using eBioscience Platinum ELISA (Thermo; San Diego, CA, USA) according to the manufacturer’s instructions.

### 2.10. Western Blot Analysis

For protein isolation, segments of the cortex and hippocampus were individually ground in a proteolysis buffer that included protease inhibitors, then chilled on ice for 10 min. The homogenates were then centrifuged at 13,000 rpm for 30 min at 4 °C. The protein content of the resultant supernatants was quantified using BCA Protein Assay kits (Sigma; 23225; St. Louis, MO, USA). For Western blotting, 50 μg of the proteins was electrophoresed on a 12% sodium dodecyl sulfate–polyacrylamide gel (SDS-PAGE) and then transferred to PVDF membranes. The membranes were blocked with TBS containing 0.05% Tween-20 (TBST) and 5% skim milk for 1 h at room temperature, followed by an overnight incubation at 4 °C with various primary antibodies [Nrf2 (proteintech; 16396-1-AP; Chicago, IL, USA), HO-1 (p-NF-κB, Cell Signaling; #3033; Danvers, MA, USA), NF-κB (Cell Signaling; #6956; Danvers, MA, USA), β-actin (Signa; A5441; St. Louis, MO, USA)] targeting specific proteins. After two washes, the membranes were treated with an appropriate HRP-linked secondary antibody for 1 h at room temperature. The enzyme activity was detected using an ECL (PerkinElmer; PK-NEL122; Hopkinton, MA, USA). Western Blotting Detection kit and visualized on X-ray films.

### 2.11. Statistical Analysis

Western blot images were quantified using Quantity One 1-D software Version 4.6.8 (BIO-RAD; Hercules, CA, USA). All data are presented as the mean ± SEM. The statistical analysis was conducted using an Analysis of Variance (ANOVA), with the Tukey–Kramer post hoc test employed to assess the significance of differences between the experimental groups. A *p*-value less than 0.05 was deemed to indicate statistical significance.

## 3. Results

### 3.1. CDDO Protects Neuron Cells

**Figure 1:**
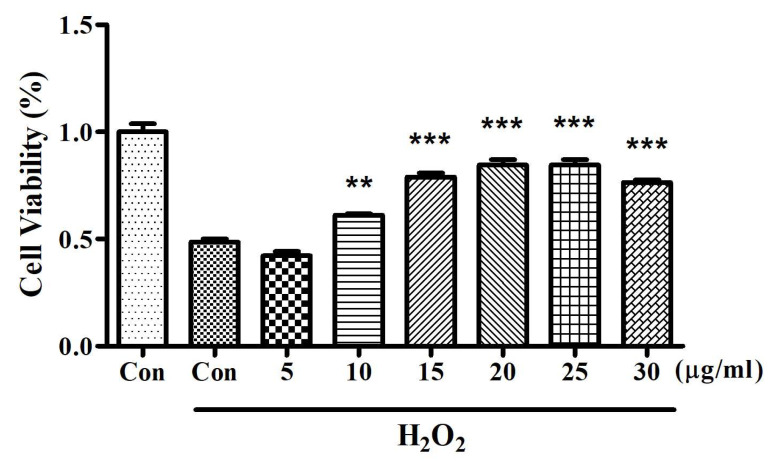
Effects of various concentrations of CDDO on cell viability in H_2_O_2_-exposed PC-12 cells. Cells were treated with H_2_O_2_ (500 μM) and different concentrations of CDDO (5, 10, 15, 20, 25, and 30 μg/mL) for 24 h. Cell viability was assessed using the MTT assay. The first bar (Con) represents control cells without treatment, while the second bar (Con + H_2_O_2_) represents cells treated with H_2_O_2_ alone. Bars indicate the mean cell viability percentage relative to the untreated control; error bars represent SEM. Statistical significance compared to the H_2_O_2_-alone-treated group is indicated by ** (*p* < 0.01) and *** (*p* < 0.001). (*n* = 6).

To determine the protective concentration of CDDO, we utilized PC-12 cells, modeling neuron cells, and conducted MTT assays to assess cell viability. This investigation compared various CDDO concentrations (0, 5, 10, 15, 20, 25, and 30 μg/mL) in the presence of 500 μM H_2_O_2_. The results revealed that cell viability in the control group treated with 500 μM H_2_O_2_ was significantly reduced compared to the untreated control group (*p* < 0.001) (Figure 1). Notably, treating the cells with 20 nM CDDO significantly enhanced their viability in the presence of 500 μM H_2_O_2_ (*p* < 0.01) (Figure 1), suggesting that while H_2_O_2_ diminished neuron cell viability, CDDO had a protective effect. Given these results, since the concentration applied to cells translates to approximately 1000 times when scaled up for use in rats, the subsequent animal experiments employed doses of 10 mg/kg and 20 mg/kg for the further study.

### 3.2. Neurological Behavioral Assessment

A neurological behavioral assessment was performed on day 7 post-SAH. In the SAH group, both the ambulation (3.09 ± 0.091) and placing/stepping reflex (1.91 ± 0.301) scores were significantly higher than those of the control group (Table 2). Although the ambulation (2.38 ± 0.140) and placing/stepping reflex (1.61 ± 0.140) scores in the low-dose CDDO group were not significantly decreased when compared with the respective values in the SAH group, their MDI score (4.00 ± 0.707) was nevertheless significantly lower when compared with that of the SAH group (5.00 ± 0.447, *p* < 0.05). On the other hand, both the ambulation (1.083 ± 0.193; *p* < 0.001) and placing/stepping reflex (0.67 ± 0.142, *p* < 0.001) scores in the high-dose CDDO group were significantly decreased when compared with those of the SAH group. Likewise, the MDI of the high-dose CDDO group (1.75 ± 0.866, *p* < 0.001) was also found to be significantly decreased compared with the SAH group (Table 2).

### 3.3. Morphological Changes in BA

**Figure 2:**
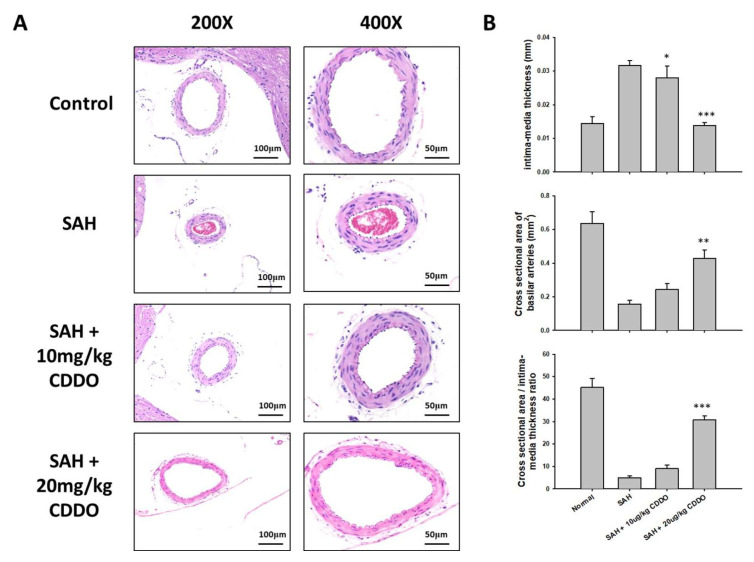
Morphometric assessment of BA in rats subjected to SAH. Representative micrographs of BA obtained from the four experimental groups in low (200×) and high (400×) magnifications are shown in (**A**). Endothelial deformation, twisting of internal elastic lamina, and smooth muscle necrosis were clearly observed in the BAs of rats subjected to SAH compared to those of the control group. A dose-dependent reversal of these effects was found upon CDDO treatment. (**B**) The BA intima-media thickness (top panel) and cross-sectional area (middle panel) in all animals were measured, and the ratios of cross-section area to intima-media thickness were calculated (bottom panel). All values are the mean ± SEM (*n* = 6). * *p* < 0.05, ** *p* < 0.01, and *** *p* < 0.001.

The microscopic analysis revealed that the basilar arteries (BAs) of rats experiencing subarachnoid hemorrhage (SAH) exhibited endothelial deformation, the distortion of the internal elastic lamina, and necrosis of smooth muscle cells. These changes were contrasted against the normal morphology observed in the control group (Figure 2A).

### 3.4. Changes in the Thickness of BA

In the control group, the basilar artery (BA) thickness was measured at 0.013 ± 0.003 mm. There was a notable increase in BA thickness in the SAH group, where it averaged 0.032 ± 0.004 mm. Treatment with low and high doses of CDDO significantly decreased BA thickness to 0.024 ± 0.005 mm (*p* < 0.05) and 0.014 ± 0.003 mm (*p* < 0.001), respectively, compared to the SAH group (Figure 2B, top panel).

### 3.5. Cross-Sectional Area Changes in BA

The mean cross-sectional area of BA was 0.637 ± 0.119 mm^2^ in the control group. In the SAH group, the BA cross-sectional area (0.178 ± 0.035 mm^2^) was reduced by 68.8% when compared with that of the control group. The BA cross-sectional area in the high-dose CDDO group was significantly increased (0.441 ± 0.093 mm^2^; *p* < 0.01) compared to the SAH group. The BA cross-sectional area in the low-dose CDDO group (0.251 ± 0.049 mm^2^) trended to be increased, but the value was not statistically different from that of the SAH group (Figure 2B, middle panel). Similar results were also obtained when the ratios of cross-sectional area to media thickness were calculated (Figure 2B, bottom panel).

### 3.6. Proliferation of Microglia and Astrocytes

**Figure 3:**
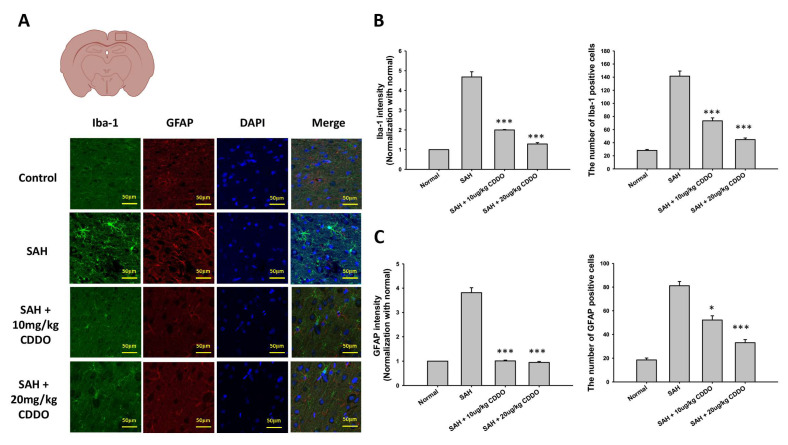
The proliferation of microglia and astrocytes in the rat brain. (**A**) Microglia and astrocytes were detected by immunofluorescence staining for Iba-1 and GFAP, respectively. Representative micrographs of Iba-1 and GFAP staining are shown for the four groups. The intensities and cell numbers of Iba-1 (**B**) and GFAP (**C**) immunofluorescence staining were quantified relative to the levels of control groups. All values are the mean ± SEM (*n* = 12). * *p* < 0.05 and *** *p* < 0.001 when compared with the SAH group.

Besides the hemorrhagic regions, there was a widespread infiltration of activated microglia into brain tissues including the brain stem, cortex, and hippocampus [11,12]. Similarly, astrocytes were observed to be activated as a response to gliosis following SAH [13]. In our research, we employed immunofluorescence staining using Iba-1 for microglia and GFAP for astrocytes to identify their presence. The induced proliferation of microglia in the rat brain was found in the SAH group (Figure 3A). A quantitative analysis of the intensity value of Iba-1 and GFAP staining set 1.0 for each in the control groups (Figure 3B). Iba-1 staining in the SAH group was substantially elevated (4.683 ± 0.456). The intensity of microglia was significantly reduced in both low-dose and high-dose CDDO groups (1.996 ± 0.041, *p* < 0.001 and 1.286 ± 0.115, *p* < 0.001, respectively). In addition, the number of Iba-1-positive cells in the SAH group (141.5 ±19.149) was significantly more than that in the control group (28.167 ± 3.656). The proliferation of microglia was significantly reduced in both low-dose and high-dose CDDO groups (73.333 ± 10.912, *p* < 0.001 and 44.667 ± 5.955, *p* < 0.001, respectively).

The results of immunofluorescence staining for GFAP mirrored those of Iba-1 staining. SAH induced the proliferation of astrocytes in the rat brain. The intensities of GFAP staining in the SAH group, low-dose CDDO group, and high-dose CDDO group were 3.813 ± 0.356, 1.010 ± 0.052, and 0.946 ± 0.066, respectively. The intensity of GFAP staining was elevated in the SAH group, and this value was significantly reduced in both low-dose and high-dose CDDO groups (both *p* < 0.001) (Figure 3B). In addition, the number of GFAP-positive cells in the SAH group (81.167 ± 8.841) was significantly more than that in the control group (18.5 ± 3.937). The proliferation of astrocytes was significantly reduced in both low-dose and high-dose CDDO groups (52.167 ± 8.819, *p* < 0.05 and 33 ± 6.419, *p* < 0.001, respectively).

### 3.7. ELISA of Proinflammatory Factors

**Figure 4:**
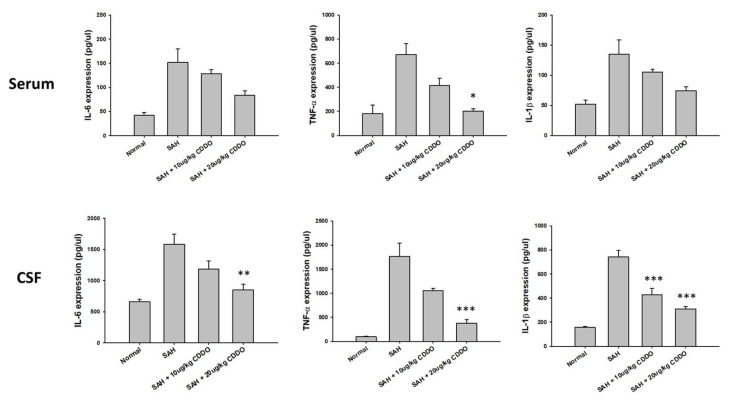
ELISA assays were conducted to quantify proinflammatory markers in the serum and CSF samples from rats 48 h following SAH. The concentrations of IL-6, TNF-α, and IL-1β were determined using kits obtained from commercial sources. Data are presented as the mean ± SEM. (*n* = 12.) * *p* < 0.05, ** *p* < 0.01, and *** *p* < 0.001 when compared with the respective SAH groups.

To explore the association between proinflammatory markers and subarachnoid hemorrhage (SAH), the concentrations of TNF-α, IL-1β, and IL-6 were measured in both CSF and serum samples using ELISA, six hours post-SAH. In both serum and CSF, expressions of IL-6, TNF-α, and IL-1β in the SAH group were significantly higher than those of the respective control groups (Figure 3). Treatment with either low- or high-dose CDDO tended to decrease the serum levels of IL-6 or IL-1β when compared with the respective values in the SAH group, but none of the differences were statistically significant. Likewise, treatment with low-dose CDDO also trended to reduce the serum levels of TNF-α when compared with that of the SAH group, but the results did not reach statistical significance. On the other hand, treatment with high-dose CDDO was found to significantly decrease the levels of serum TNF-α (*p* < 0.05). In CSF, the expressions of IL-6, TNF-α, and IL-1β in the high-dose CDDO group were significantly decreased compared with those of the respective SAH groups (*p* < 0.01, *p* < 0.001, and *p* < 0.001, respectively), but only the expression of IL-1β was significantly decreased in the low-dose CDDO group (*p* < 0.001) (Figure 4). All of the aforementioned results appeared to show dose-dependent effects of CDDO.

### 3.8. DNA Damage of Neuron Cells

Apoptosis serves as a secondary mechanism in response to DNA damage, aiming to safeguard multicellular organisms by eliminating damaged cells [14]. In our research, immunofluorescence staining was employed to assess neuronal DNA damage. The staining for NeuN and cleaved caspase-3 was performed to assess the impact of CDDO on neuronal survival and apoptosis within brain tissues. NeuN is a specific marker for neurons, commonly employed for neuronal identification and quantification. Through NeuN staining, we aimed to evaluate the protective effects of SAH and CDDO on neurons.

**Figure 5:**
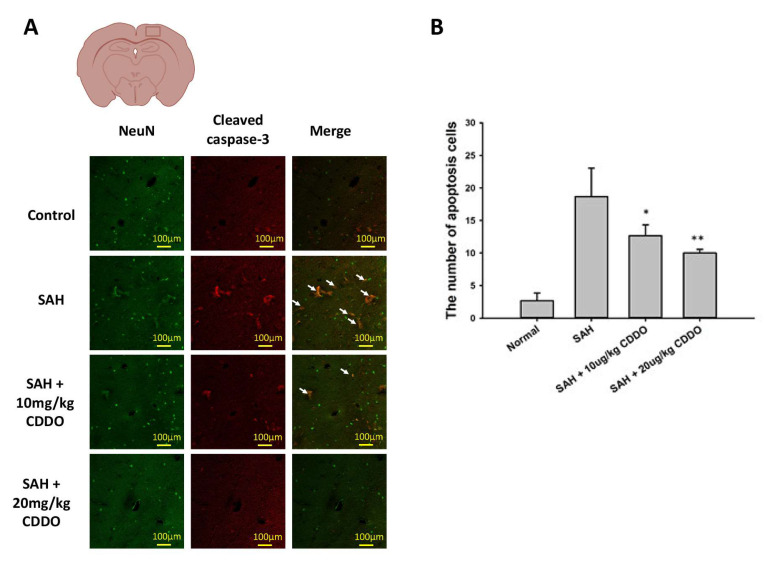
DNA damage of neuron cells in the rat brain determined by immunofluorescence staining for NeuN and cleaved caspase-3. (**A**) Representative micrographs of immunofluorescence staining are shown for the four groups. The white arrows indicate cells with positive double staining, showing co-expression of NeuN and cleaved caspase-3. (**B**) The number of neuronal cells with DNA damage from immunofluorescence staining in the four groups was measured. All values are mean ± SEM (*n* = 12). * *p* < 0.05 and ** *p* < 0.01 when compared with the SAH group.

On the other hand, cleaved caspase-3 is an indicator of cellular apoptosis, and its staining was used to assess the extent of apoptosis. The quantitative evaluation of double immunofluorescence staining for NeuN showed an average of 2.50 ± 1.37 in the control group (Figure 5). Following SAH, there was a significant increase in neuronal DNA damage in the rat brain, with double staining values rising markedly to 21.16 ± 7.33 in the SAH group, and the DNA damage of neuron cells was significantly reduced in both low-dose and high-dose CDDO groups (15.00 ± 3.03, *p* < 0.05 and 10.16 ± 2.99, *p* < 0.01, respectively) when compared with the SAH group (Figure 5).

### 3.9. Western Blot Analysis

**Figure 6:**
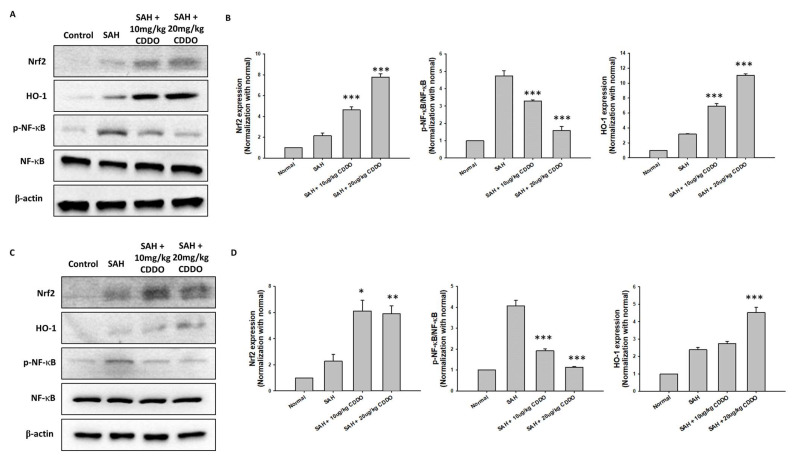
Representative results of the Western blot analysis to show the effect on the levels of Nrf2, HO-1, p-NF-κB, and NF-κB protein in the cortex (**A**,**B**) and in the hippocampus (**C**,**D**) of rats at 48 h following SAH. The Nrf2, HO-1, and p-NF-κB/NF-κB expression levels were normalized using the internal control (β-actin). All values are the mean ± SEM (*n* = 12). * *p* < 0.05, ** *p* < 0.01, and *** *p* < 0.001 when compared with the SAH group.

The altered expression of Nrf2, HO-1, and NF-κB has been shown in various brain injuries. Therefore, expressions of these proteins and phosphorylated NF-κB (p-NF-κB) in the cortex and hippocampus were separately assessed at 48 h after SAH. Expression levels of these proteins were all significantly increased in the SAH group when compared with the respective control groups (Figure 6). In the cortex (Figure 6A,B), the levels of Nrf2 and HO-1 were significantly increased (*p* < 0.001) while the level of p-NF-κB/NF-κB was significantly decreased (*p* < 0.001) in both the low-dose and high-dose CDDO groups when compared with the SAH group. Similar results were also obtained in the hippocampus (Figure 6C,D). The levels of Nrf2 and HO-1 were also significantly increased (*p* < 0.01 and *p* < 0.001, respectively) and the p-NF-κB/NF-κB levels were significantly decreased (*p* < 0.001) in the high-dose CDDO group. In the low-dose CDDO group, the Nrf2 level (*p* < 0.05), but not the HO-1 level, was also significantly increased, while the p-NF-κB/NF-κB level was significantly decreased (*p* < 0.001) in the hippocampus.

## 4. Discussion

The induction of brain inflammation following SAH involves a complex interplay of various factors. Inflammatory cytokines, reactive oxygen species, and the disruption of the blood–brain barrier (BBB) are all thought to contribute to the inflammatory response seen in SAH [15]. Numerous inflammatory cytokines such as IL-1, IL-6, and TNF-α are closely linked to brain injury in rats [16]. These cytokines are implicated in the pathological processes that lead to blood–brain barrier (BBB) disruption and brain edema, both of which are hallmarks of clinical and experimental SAH [17,18]. Previous studies have demonstrated that blocking IL-1β can reduce early brain injury and enhance BBB integrity following SAH [19]. In addition, the levels of TNF-α and IL-1β mRNA expression in injured brain tissues of SAH rats were significantly higher than in the control group, indicating the involvement of these cytokines in the inflammatory response. Consistent with these findings, elevated expressions of IL-6, TNF-α, and IL-1β were also observed in the serum and CSF samples of SAH rats (Figure 3) in the present study. Treatment with CDDO decreased the expressions of these cytokines, mitigated the morphological changes in the BA (Figure 1), and improved the behavioral outcomes (Table 2).

SAH induces the proliferation and activation of microglia and astrocytes, resident immune cells in the central nervous system. Activated microglia and astrocytes undergo morphological changes, and they may aggravate SAH-induced brain injury by further secreting inflammatory factors [20,21,22], such as cytokines, chemokines, and complement proteins. On the other hand, the inhibition of microglia and astrocyte activation attenuates brain injury following SAH. Similar to these data, the proliferation of microglia and astrocytes was also noted in the brains of SAH rats, and treatment with CDDO inhibited the proliferation of these cells (Figure 2).

In the present study, increased apoptosis of neuronal cells was seen in the brain of SAH rats, and treatment with CDDO significantly attenuated this process (Figure 4). Neuronal apoptosis resulting from SAH can have significant implications for BBB dysfunction and inflammation. It can lead to brain damage and the activation of the inflammatory cascades and oxidative stress pathways, which in turn can further contribute to the development of brain edema and the occurrence of cerebral vasospasm following SAH. Furthermore, studies have shown that the inhibition of caspase-3, a protein involved in apoptosis, can reduce neuron loss and brain edema [23]. These results suggest that inhibiting neuron apoptosis may be an effective therapeutic strategy to lessen brain damage and improve neurologic outcomes.

The present study also demonstrated that treatment with CDDO increased the expression of Nrf2 and HO-1 in the cortex and hippocampus of rats when compared with SAH rats without treatment, whereas the levels of p-NF-κB were significantly reduced upon the same treatment. Nrf2 is a transcription factor that plays a crucial role in cellular defense against oxidative stress by inducing the expression of cytoprotective and antioxidant genes [24]. CDDO is an activator of Nrf2, and it has been reported that CDDO derivatives may attenuate cerebral ischemic injury by promoting microglia/macrophage polarization toward the M2 phenotype in mice [25]. In addition, CDDO was found to induce HO-1 and regulate the Nrf2-mediated oxidative stress response pathway [26]. NF-κB is a nuclear transcription factor that acts as a key regulator of both the inflammatory response and cell death [27,28]. It has been shown that NF-κB induces the expression of various proinflammatory genes. In this study, the NF-κB pathway was activated by SAH. Subsequently, it may lead to the observed microglia and astrocyte proliferation, proinflammatory cytokine production, vasospasm, and neuronal cell death. In our study, CDDO attenuates vasospasm and neuronal cell apoptosis following SAH. However, related research by Tsai TH et al. on a second-generation semisynthetic oleanane triterpenoid, RTA 408, addresses its effects on SAH-induced delayed cerebral vasospasm and secondary brain injury, suggesting a broader applicative scope for these compounds in vascular and neuro-inflammatory conditions [29]. The primary distinction between CDDO and RTA 408 lies in the variation of their functional groups, indicating that the core structure itself possesses the capacity to mitigate SAH-induced physiological changes. This insight suggests that the fundamental backbone of these molecules could serve as a template for the development of new therapeutic agents targeting SAH in future research endeavors. Related research by Cheng L et al. on the Nrf2 activator properties of oleanane triterpenoids, such as CDDO, has demonstrated their role in vascular diseases [30]. Furthermore, another study by Tran TA et al. explored the CDDO-methyl ester’s capacity to modulate microglial activities and inhibit TNF production, providing significant neuroprotection. This demonstrates the compound’s potential in neuroinflammatory and neurodegenerative diseases by directly influencing key mechanisms of neurodegeneration [31]. Finally, a recent manuscript by Lu CC et al. illustrates how CDDO regulates central and peripheral sensitization to effectively reduce post-herpetic neuralgia by targeting specific neural signaling pathways. This finding not only underlines its role in pain control but also reinforces its broader implications in neural protection [32]. These studies collectively affirm the capability of the CDDO core structure to both suppress inflammation and safeguard neural cells, highlighting its potential for further development in therapies aimed at conditions involving both inflammation and neural damage.

## 5. Conclusions

In summary, impaired behavioral responses, morphological changes in the BA, the activation of microglia and astrocytes, increased apoptotic cells in the brain, enhanced expressions of proinflammatory factors, and altered expressions of nuclear factors were all seen in rats subjected to SAH. Treatment with CDDO significantly attenuated all of the aforementioned changes. The results presented herein suggest that CDDO may inhibit the production of p-NF-κB and enhance the Nrf2/HO-1 pathway.

## Figures and Tables

**Table 1:** Motor function examination.

Motor	Behavior	Score
Ambulation	Normal (symmetric and coordinated)	0
	Toes flat under the body while walking with ataxia	1
	Knuckle walking	2
	Movement in lower extremities but unable to knuckle walk	3
	No movement, dragging lower extremities	4
Placing/stepping reflex	Normal (coordinated lifting and placing response)	0
	Weak response	1
	No stepping	2

**Table 2:** Behavioral assessment.

Treatment	Ambulation	Placing/Stepping Reflex	MDI
Control	0	0	0
SAH	3.09 ± 0.091	1.91 ± 0.301	5.00 ± 0.447
SAH + 10 mg/kg of CDDO	2.38 ± 0.140	1.61 ± 0.140	4.00 ± 0.707 *
SAH + 20 mg/kg of CDDO	1.083 ± 0.193 ***	0.67 ± 0.142 ***	1.75 ± 0.866 ***

Neurological function was evaluated at 7 days after SAH by motor function and total scores of the motor deficit index (the sum of ambulation and placing/stepping reflex scores). MDI, motor deficit index. Results are expressed as the mean ± SEM (*n* = 6). * *p* < 0.05; *** *p* < 0.001, compared with the SAH group.

## Data Availability

Data is contained within the article.
